# Supplementary material for: Glucuronidated Metabolites of Bisphenols A and S Alter the Properties of Normal Urothelial and Bladder Cancer Cells
Source: Int J Mol Sci. 2022 Oct 25;23(21):12859. doi: 10.3390/ijms232112859 (PMC9656169; doi:10.3390/ijms232112859)
Supplement: Supplementary file 1 [file ijms-23-12859-s001.zip › ijms-1917814-supplementary.pdf]

SUPPLEMENTARY FIGURES

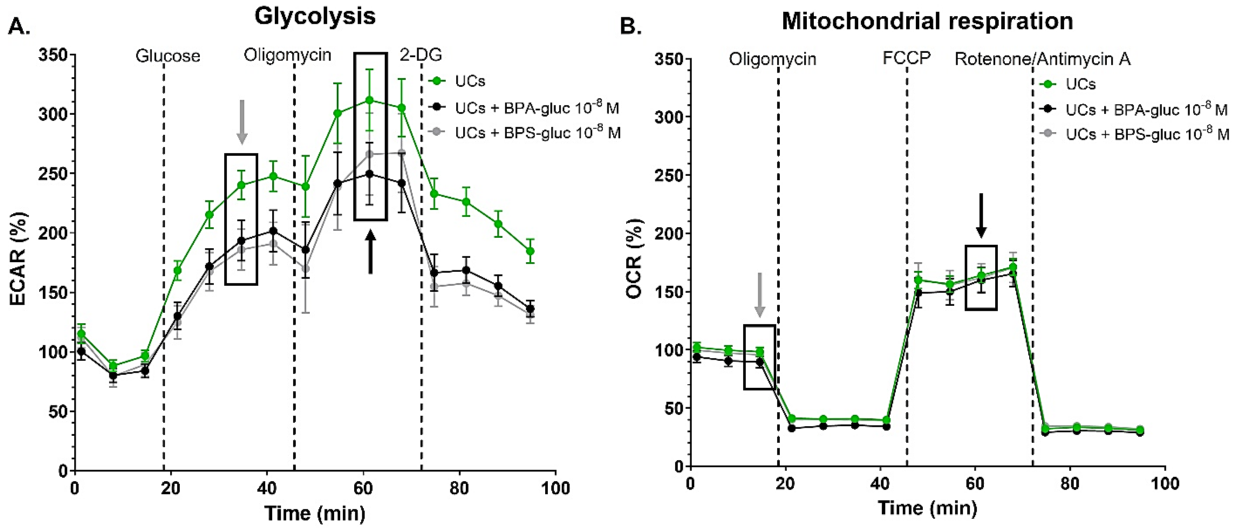

Figure S1. Impact of BPA-gluc and BPS-gluc on the glycolytic and mitochondrial metabolism of normal urothelial cells (UCs). (**A**) The glycolytic metabolism was established by the sequential injections of glucose, oligomycin and 2-DG. Analyses in Figure 1A-B were performed using measure #6 (gray arrow) for basal glycolysis and measure #10 (black arrow) for maximal glycolytic capacity. (**B**) The mitochondrial respiration was established by sequential injections of oligomycin, FCCP and the combination of rotenone and antimycin A. Analyses in Figure 1C-D were performed using measure #3 (gray arrow) for basal mitochondrial respiration and measure #10 (black arrow) for maximal mitochondrial respiration. Data are displayed as percentages of controls (i.e., untreated condition) (n = 3, N = 4). The baseline (100%) was established before the first injection, namely before glucose injection for the glycolytic capacity and before oligomycin injection for the mitochondrial respiration.

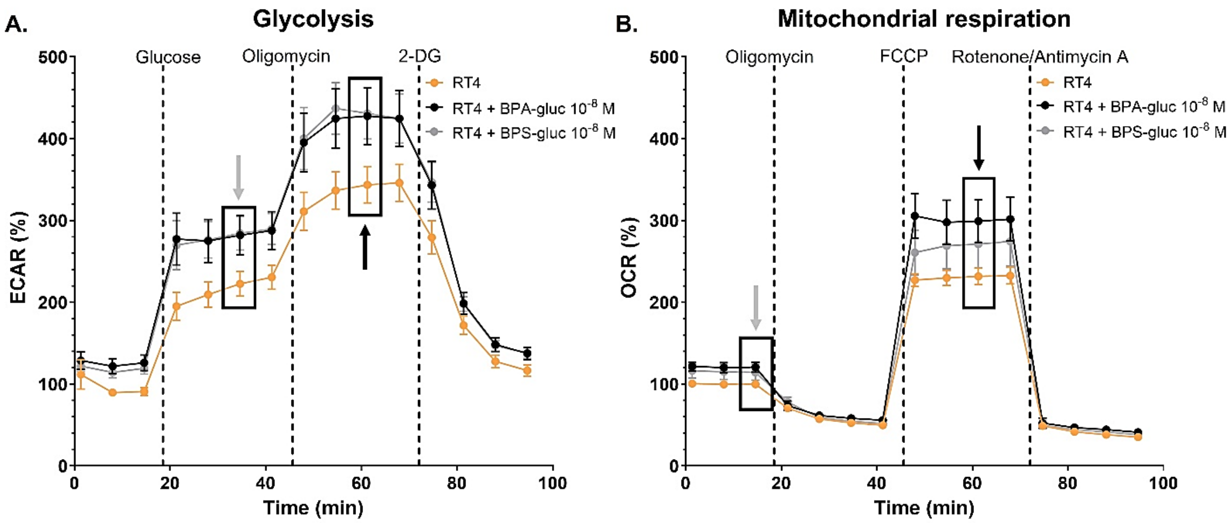

Figure S2. Impact of BPA-gluc and BPS-gluc on the glycolytic and mitochondrial metabolism of RT4 non-invasive bladder cancer cells. (**A**) The glycolytic metabolism was established by the sequential injections of glucose, oligomycin and 2-DG. Analyses in Figure 2A-B were performed using measure #6 (gray arrow) for basal glycolysis and measure #10 (black arrow) for maximal glycolytic capacity. (**B**) The mitochondrial respiration was established by sequential injections of oligomycin, FCCP and the combination of rotenone and antimycin A. Analyses in Figure 2C-D were performed using measure #3 (gray arrow) for basal mitochondrial respiration and measure #10 (black arrow) for maximal mitochondrial respiration. Data are displayed as percentages of controls (i.e., untreated condition) (n = 3, N = 3). The baseline (100%) was established before the first injection, namely before glucose injection for the glycolytic capacity and before oligomycin injection for the mitochondrial respiration.

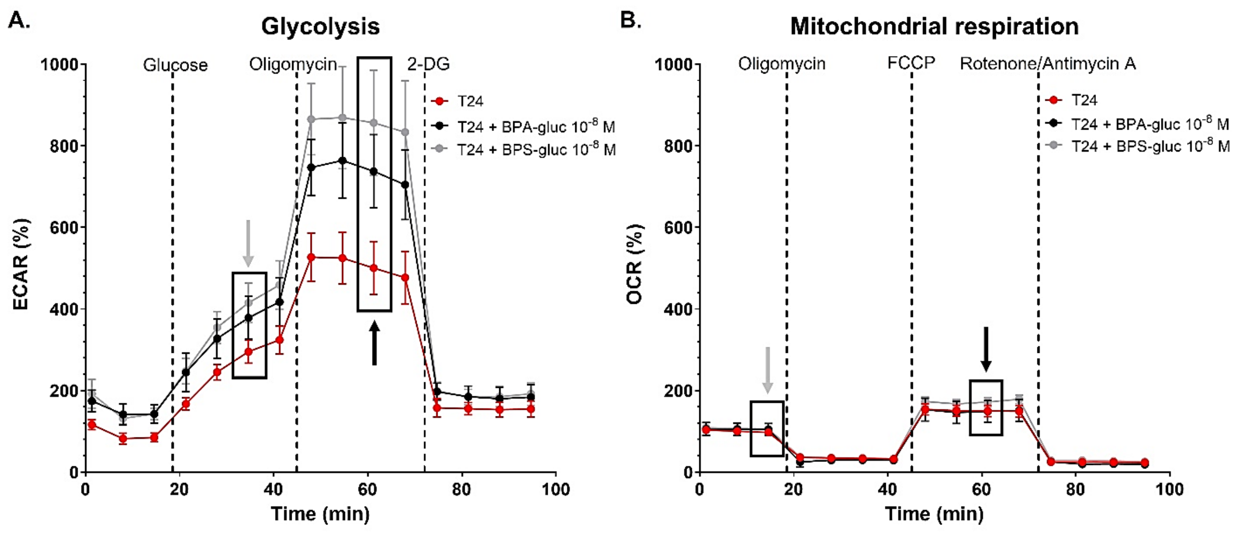

Figure S3. Impact of BPA-gluc and BPS-gluc on the glycolytic and mitochondrial metabolism of T24 invasive bladder cancer cells. (**A**) The glycolytic metabolism was established by the sequential injections of glucose, oligomycin and 2-DG. Analyses in Figure 3A-B were performed using measure #6 (gray arrow) for basal glycolysis and measure #10 (black arrow) for maximal glycolytic capacity. (**B**) The mitochondrial respiration was established by sequential injections of oligomycin, FCCP and the combination of rotenone and antimycin A. Analyses in Figure 3C-D were performed using measure #3 (gray arrow) for basal mitochondrial respiration and measure #10 (black arrow) for maximal mitochondrial respiration. Data are displayed as percentages of controls (i.e., untreated condition) (n = 3, N = 3). The baseline (100%) was established before the first injection, namely before glucose injection for the glycolytic capacity and before oligomycin injection for the mitochondrial respiration.
